# Agreement of zero-heat-flux thermometry with the oesophageal and tympanic core temperature measurement in patient receiving major surgery

**DOI:** 10.1007/s10877-023-01078-2

**Published:** 2023-10-04

**Authors:** Hao Liang, Jing-Yan Wang, Yan Liang, Xin-Feng Shao, Yan-Ling Ding, Hui-Qun Jia

**Affiliations:** 1 Department of Anesthesiology, The Fourth Hospital Of Hebei Medical University, Shijiazhuang, Hebei China; 2 Department of ENT, Affiliated Hospital Of Hebei University, Baoding, Hebei China; 3 Department of Obstetrics, The NO.1 Central Hospital Of Baoding City, Baoding, Hebei China; 4 Department of Anesthesiology, The NO.1 Central Hospital Of Baoding City, Baoding, Hebei China

**Keywords:** Zero heat flux, Hypothermia, Core temperature measurement, Major surgery, General Anesthesia

## Abstract

To identify and prevent perioperative hypothermia, most surgical patients require a non-invasive, accurate, convenient, and continuous core temperature method, especially for patients undergoing major surgery. This study validated the precision and accuracy of a cutaneous zero-heat-flux thermometer and its performance in detecting intraoperative hypothermia. Adults undergoing major non-cardiac surgeries with general anaesthesia were enrolled in the study. Core temperatures were measured with a zero-heat-flux thermometer, infrared tympanic membrane thermometer, and oesophagal monitoring at 15-minute intervals. Taking the average value of temperature measured in the tympanic membrane and oesophagus as a reference, we assessed the agreement using the Bland-Altman analysis and linear regression methods. Sensitivity, specificity, and predictive values of detecting hypothermia were estimated. 103 patients and one thousand sixty-eight sets of paired temperatures were analyzed. The mean difference between zero-heat-flux and the referenced measurements was -0.03 ± 0.25 °C, with 95% limits of agreement (-0.52 °C, 0.47 °C) was narrow, with 94.5% of the differences within 0.5 °C. Lin’s concordance correlation coefficient was 0.90 (95%CI 0.89–0.92). The zero-heat-flux thermometry detected hypothermia with a sensitivity of 82% and a specificity of 90%. The zero-heat-flux thermometer is in good agreement with the reference core temperature based on tympanic and oesophagal temperature monitoring in patients undergoing major surgeries, and appears high performance in detecting hypothermia.

## Introduction

Physiological thermoregulation is a crucial factor in maintaining homeostasis in the body [1]. Human thermoregulation maintains the core temperature in a relatively narrow and safe range by balancing the production and consumption of body heat [2]. Affected by protopathy, surgery, and anaesthesia, the balance of thermoregulation is broken, and patients’ core temperature often fluctuates substantially during the perioperative period [3]. In addition to infectious fever, hypothermia is the most common thermos dysfunction during surgery [4]. Perioperative hypothermia can lead to adverse effects like coagulopathy, myocardial ischemia, delayed drug metabolism, prolonged recovery, shivering, and surgical site infection [3]. For anaesthesia professionals, there is a need for a continuous and reliable, particularly easily accessible method of core temperature during the management of patients.

Core temperature monitoring is especially applicable to patients under general anaesthesia. In addition to detecting hypothermia and guiding the active warming equipment, it can identify malignant hyperthermia early. However, an ideal temperature measurement approach has not been established. The pulmonary artery is recognized as the most accurate route to measure core body temperature, which is greatly restricted considering its applicability as an invasive procedure [5]. Ideal core body temperature monitoring contains the properties of being noninvasive, accurate, stable, reproducible, convenient, and inexpensive [6, 7].

The thermometer based on the zero heat flux (ZHF) principle, first described by Fox [8] and modified from 3 M (USA), can be an option for ideal core temperature monitoring. The sensor consists of an insulator patch and a servo-controlled electric heater. In this process, the sensor is placed on the skin of the forehead creating an isothermic tunnel from deep tissue to the forehead. The heater’s self-regulation keeps the skin and insulator at the same temperature, which is identified as the core temperature when the heat flowing is in equilibrium from the core to the covered forehead [4].

A validation study to evaluate the zero-heat-flux thermometer in major surgery patients is significant. Because these patients are more prone to severe temperature fluctuation, they often need multiple ways of thermal interventions [9, 10]. The tympanic infrared thermometer and oesophagus thermocouple have been proven to be close to pulmonary artery temperature when probes are inserted under proper positioning [11, 12] and are commonly used in perioperative practice [4, 13]. Specifically, to accurately present core body temperature in a non-invasive manner and prevent errors from selection of the measurement site [4], we considered the mean of the esophagus and eardrum measurements as the referenced core temperature [14]. We aimed to determine the accuracy and precision of the ZHF thermometer compared with the referenced core temperature and further estimate the performance in the detection of intraoperative hypothermia and hyperthermia.

## Methods

The study protocol was registered at the Chinese Clinical Trial Registry (ChiCTR2200057548) after obtaining the approval of the local ethics committee. The study complied with the declaration of Helsinki, and all participants provided informed consent.

Adult patients with the American Society of Anesthesiologists (ASA) Physical Status classification of I–III, who was scheduled for surgery with an estimated time greater than 1 h, were recruited in the study between January 2022 and February 2023. Participants were excluded when encountering any contraindication or influence on temperature measurement(e.g. canal infection, head and neck surgery, hemodynamic instability, intraoperative use of transdermal preparations, continuous mechanical ventilation after operation).

After entering the operating room, a disposable, continuous, non-invasive ZHF sensor was attached to the forehead. Connect the sensor with the ZHF thermometry(3 M Company, St Paul, MN, USA), and record the core temperature after running for 3–5 min to equilibrate. Before monitoring with the tympanic membrane thermometer (Welch Allyn Braun Pro4000 Thermoscan, Welch Allyn, Skaneateles Falls, NY, USA), clean the external ear canal with an alcohol cotton swab. The anesthesiologist gently pulled the patient’s auricle back to straighten the external auditory canal and inserted the probe into the ear canal for measurement.

After anaesthesia induction, with the assistance of a visual laryngoscope, an oesophagal thermometer probe (Philips Medical Systems Model 21,075 A, Andover, MA) was placed through one nostril. The depth was determined by standing height according to the Mekjavic formula [15]. Continuous temperature data of the forehead and oesophagal measurement were downloaded from the monitor storage system. The infrared tympanic temperature were performed and recorded every 15 min from the beginning of anaesthesia until the end. The measurements of tympanic temperature were fixed on the same ear and the averaged values of two consecutive measurements were taken for the final analysis. All measurements were performed by the anesthesiologist who had received standardized training for the three thermometers. The personnel who read the stored electronic data of forehead and esophageal temperature at the corresponding moment adopted double-entry verification and avoided the participation of clinicians who collect eardrum temperature.

All patients underwent standardized vital signs monitoring during the perioperative period. The attending anesthesiologist in charge determines the management of anaesthetic, respiration, fluid, and thermal preservation. So was the choice of thermal preservation. Active warming methods include forced-air warming (WarmTouch 5300 A, SOMA TECH INTL, Bloomfield, CT, USA) and intravenous fluid warming devices (Flotherm QW618, Keewell, Foshan, Guangdong, P.R.China). Care of passive warming methods contains the cotton blanket and surgical drape. The ambient temperature was controlled at 20–24 °C.

The primary outcome measure was agreement between the ZHF monitor temperature and the reference core temperature during general anesthesia. We determined the mean value of oesophagal temperature and tympanic membrane temperature as patients’ core temperature. Assuming that a difference of ≤ 0.5 °C would be clinically acceptable. The accuracy of the ZHF thermometer was evaluated by Bland-Altman analysis with random effects model [16, 17] and the proportion of bias within ± 0.5 °C. The Bland-Altman method for repeated measurement data constructed the patient, baseline measurement and collects time as the random factors and estimated the variance after adjusting for multiple variables. Mean difference, standard deviation (SD) and limits of agreement(LOA)of the bias were calculated from the same model. Since the lack of unified standards for power calculation, a sample size of 100 patients was considered sufficient according to previous studies [18, 19]. Lin’s concordance correlation coefficient (CCC) and linear regression model for repeated measurements were performed to summarize the association between the two methods.

The second outcome measure was the performance of the ZHF thermometer in detecting hypothermia and hyperthermia, which was evaluated by calculating sensitivity, specificity, and predictive values. Hypothermia was defined as the reference core temperature of < 36.0 °C, and hyperthermia was defined as a core temperature > 37.5 °C.Data analyses were performed with MedCalc software 20.111 (MedCalc Software Ltd, Ostend, Belgium), GraphPad Prism 9.0 software (GraphPad Software, San Diego, California, USA), and SPSS Statistics V26.0 software (IBM Corp., Armonk, NY, USA). 95% confidence interval(CI) and limits of agreement for the results were presented when appropriate. The statistical significance was set at 0.05.

## Results

We finally enrolled 103 patients. Three of them were excluded from the study, 2 patients had surgery for less than 1 h, and 1 patient whose oesophagal temperature monitoring was interrupted by a surgery problem. Consequently, 1068 pairs of qualifying temperature data were included in the analysis. Among the involved patients, the average age was 60.2 ± 12.8 years, 60.2% were males, and the mean body mass index (BMI) was 25.5 ± 4.6 kg/m2. The types of surgery included thoracic surgery in 26 (25.2%) patients, orthopaedic surgery in 31(30.1%) patients, urological surgery in 25(24.3%) patients, and general surgery in 21 (20.4%) patients. 38 (36.9%) patients accepted intraoperative active warming methods. The demographic and surgical features are summarized in Table 1.

**Table 1:** Patient demographics and anesthesia/surgery data

Variables	Value, proportion(%);or Median
Age, mean ± SD (years)	60.2 ± 12.8
Gender	
Male	62(60.2%)
Female	41(39.8%)
BMI (kg/m^2^, mean ± SD)	25.5 ± 4.6
Type of surgery	
Thoracic Surgery	26(25.2%)
Orthopedic Surgery	31(30.1%)
Urological Surgery	25(24.3%)
General Surgery	21(20.4%)
Patient warming	
Intraoperative passive warming	65(63.1%)
Intraoperative active warming	38(36.9%)

*SD* standard deviation, *BMI* body mass index

**Fig. 1:**
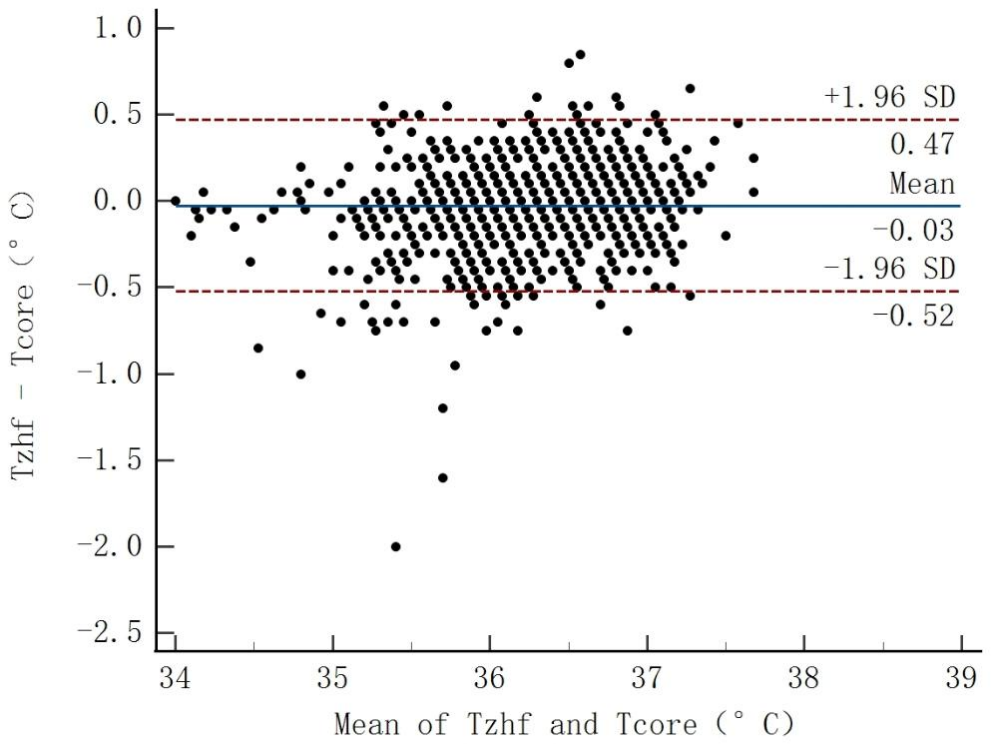
Bland-Altman plot for zero-heat-flux thermometer versus reference core temperature. *T*_*zhf*_ temperature of zero-heat-flux thermometer, *T*_*core*_ the mean temperature of tympanic membrane and oesophagal, *SD* standard deviation

**Fig. 2:**
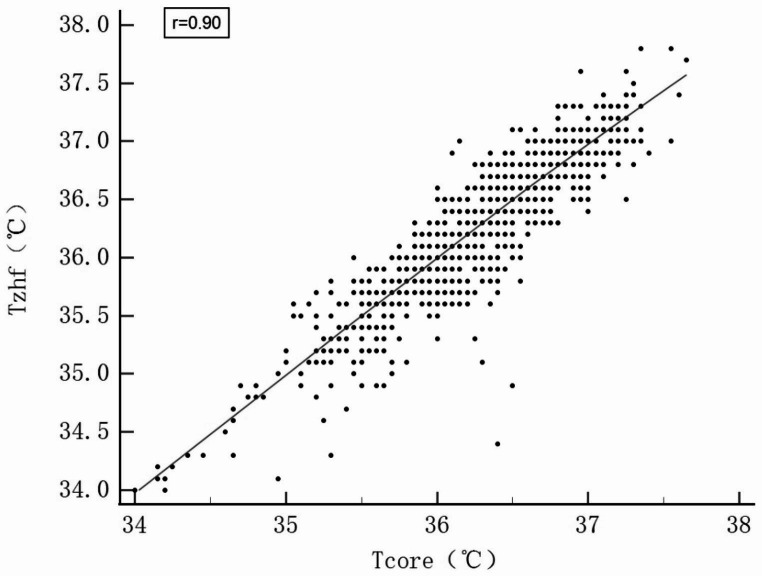
The regression line from concordance analysis shows perfect agreement between the zero-heat-flux thermometer (T_zhf_) and the reference core temperature (T_core_). *T*_*zhf*_ temperature of zero-heat-flux thermometer, *T*_*core*_ the mean temperature of tympanic membrane and oesophagal

As shown in Fig. 1, the ZHF thermometer and the reference measurements agreed well overall. The bias was − 0.03 °C(95%CI, -0.04 to -0.01)with an SD of 0.25. The upper LOA for the ZHF sensor was 0.47 °C, and the lower LOA was − 0.52 °C. Moreover, the 95% CI calculated from the LOA ranged from − 0.55 °C to 0.49 °C (Table 2). The proportion of measurements within 0.5 °C of the reference temperature was 95.41% (95%CI, 93.98–96.51%). CCC resulted in 0.90 (95%CI, 0.89–0.92), indicating a strong agreement between the compared temperatures (Fig. 2).

**Table 2:** Agreement Between Zero-heat-flux Thermometer and Reference Core Temperature

Measure	T_zhf_ Versus T_core_
Bias (mean ± SD)	-0.03 ± 0.25
95% limits of agreement Lower limit (95% CI) Upper limit (95% CI)	-0.52 (-0.55 to -0.50) 0.47 (0.44–0.49)
Proportion of difference within 0.5 °C(95% CI)	95.41% (93.98–96.51%)
CCC(95% CI)	0.90 (0.89–0.92)

*SD* standard deviation, *CI* confidence interval, *CCC* concordance correlation coefficient

The performance of the ZHF thermometer for detecting hypothermia and hyperthermia is shown in Table 3. The sensitivity of the ZHF monitor in detecting hypothermia was 82.0%, with a specificity of 90.0%. The positive and negative predictive values (PPV, NPV) were 75.0% and 93.0%, respectively. Our findings suggest that the SponOn sensor showed high reliability in diagnosing hypothermia. A total of 3 patients were detected with hyperthermia, with 4 sets of measurements affected by the care of active warming, and the ZHF thermometer had lower sensitivity (50%) and PPV (29%) in the study.

**Table 3:** Sensitivity, specificity, positive and negative predictive values for the detection of hypothermia and hyperthermia of temperature measured with the zero-heat-flux thermometer

	Sensitivity (95% CI)	Specificity (95% CI)	PPV (95% CI)	NPV (95% CI)
Detection of hypothermia	0.82(0.78–0.87)	0.90(0.89–0.92)	0.75(0.70–0.79)	0.93(0.92–0.95)
Detection of hyperthermia	0.50(0.15–0.85)	0.99(0.99-1)	0.29(0.08–0.64)	0.99(0.99-1.00)

*PPV* positive predictive value, *NPV* negative predictive value, *CI* confidence interval

## Discussion

In this study with patients undergoing major surgeries, we compared the ZHF thermometer with commonly used clinical core temperature measurements, including the tympanic membrane thermometer and oesophagal temperature probe. To our knowledge, this is the first time to use the mean value of the two non-invasive core temperature monitors as the reference temperature. Data analysis has shown that the ZHF sensor was strongly correlated with the referenced temperature and performed a high accuracy for diagnosing intraoperative hypothermia.

95.41% of ZHF temperatures were within ± 0.5 °C of the reference temperature from the tympanic membrane and distal oesophagal measurements. Meanwhile, the CCC statistics and Bland Altman analysis demonstrated almost perfect agreement. Even though the 95% CI slightly exceeded 0.5 °C as predetermined, our results are consistent with previous studies that ZHF temperature measurement has minor bias [20, 21]. However, in this case, bias is not as important as precision. It usually represents a calibration error because of the adjustment by the software [19]. Most studies defined a limit of agreement within ± 0.5 °C between thermometers as clinically acceptable [19–22]. To avoid the sampling error of LOA, we calculated the 95% CI, which can improve the accuracy of consistency evaluation [23, 24].

Additional results showed that the ZHF monitor could detect hypothermia with a sensitivity of 0.82 and a specificity of 0.90. Considering that the most common thermal disturbance in surgical patients is accidental hypothermia [4], our results demonstrate the clinical implication of the continuous noninvasive thermometer. To evaluate the performance of the ZHF system in diagnosing hyperthermia, we performed an analysis using 37.5 centigrade as the reference [25]. Affected by the low incidence of perioperative hyperthermia [25] and the excessive sample size(3 patients, 4 sets)of fever in this study, our results do not have much clinical significance on the validity of the ZHF monitor in hyperthermia.

The accuracy and precision of any temperature monitoring depend on the equipment and the measurement site [4, 26]. There are specific differences in tissue temperature from different parts [14]. Pulmonary artery catheter has long been the most reliable measure of core body temperature [27]. To avoid the risk of being highly invasive from pulmonary artery catheters [28], studies have confirmed that the infrared ear monitor and oesophagal temperature probe could substitute for it [26]. A systematic review estimated sixteen high-quality studies to determine the agreement of the ZHF thermometer between different core temperature measurements from multiple clinical circumstances [29]. The mean bias was 0.03 °C, but the LOA was wide, which may underestimate the precision in our opinion. Considering the influence of metabolism, tissue conduction and blood flow on core temperature, Taylor et al. concluded that in the study of temperature regulation and dynamic changes, deep-body temperature measurements must be selected from the central blood volume or more sites which reliably track the temperatures [14]. This comparison proposes the mean value of tympanic temperature and oesophagal temperature as a reference, which can more objectively reflect the core temperature.

Previous studies [30] and our clinical experience show that the measurement site and environment also affect the accuracy of ZHF measurement. The system may be affected by the external temperature change and airflow near the sensor, and for example, there is an air conditioning outlet above the patient’s head. We suggest the patient’s head be isolated with a surgical sheet when using the ZHF thermometer in an operating room. Considering that the accuracy of ZHF measurement depends on the equilibrated isothermal channel between the skin and deep tissue under the temperature sensor, it is essential to connect and place the sensor following the operation manual.

In addition to the properties exhibited statistically, the ZHF thermometer performs outstanding advantages for clinical applications, which make it the ideal form of core temperature monitoring in the perioperative period. Compared with tympanic and oesophagal temperature measurement, it is an entirely non-invasive, continuously monitored device that causes no discomfort to patients and is convenient to perform [31]. The data acquired are minimally affected by the operator. In addition, it can be used to monitor core temperature for the patients awakened continuously [30].

The study has some limitations. The research design lacks attention to blinding-related issues. The anesthesiologist who recorded the infrared tympanic measurements was aware of the monitoring of the ZHF and esophageal temperature during the surgery, which could have affected the results. The operations observed in this study are non-cardiac. The range and extent of change in core body temperature in cardiac surgery are more dramatic, and the influencing factors are more complicated [32, 33]. The accuracy of the ZHF system in cardiovascular surgery requires further validation. Affected by the low incidence and the small sample size of perioperative hyperthermia, we cannot comment on the performance of the ZHF sensor in diagnosing hyperthermia. Finally, the study evaluated the forehead core temperature with only one model of ZHF equipment.The performance may be different from another brand of ZHF thermometry.

To conclude, our study demonstrates that ZHF thermometers have a good agreement with the reference core temperature based on tympanic and oesophagal temperature monitoring in general anaesthesia patients undergoing major non-cardiac surgeries. We further confirmed the reliability of the ZHF system in detecting hypothermia. However, the performance of the ZHF thermometer in patients with hyperthermia and cardiac surgery needs further evaluation.

## Data Availability

Data used during the current study is available from the corresponding author on reasonable request.
